# Supplementary material for: MetaRibo-Seq measures translation in microbiomes
Source: Nat Commun. 2020 Jun 29;11:3268. doi: 10.1038/s41467-020-17081-z (PMC7324362; doi:10.1038/s41467-020-17081-z)
Supplement: Supplementary file 10 — Supplementary Data 7 [file 41467_2020_17081_MOESM10_ESM.zip › File2/Confidence_High_Taxonomy/108994_out.krona.html]

Javascript must be enabled to view this page.

members
magnitude
magnitudeUnassigned
count
unassigned
taxon
rank

108994\_out

25

25
superkingdom
2

1239
phylum
25

class
186801
25

order

SRS023526\_contig\_number\_29456SRS055017\_contig\_number\_contig-100\_10234.167372SRS063127\_contig\_number\_contig-100\_4171.4172SRS077849\_contig\_number\_contig-100\_20715.56905SRS100021\_contig\_number\_contig-100\_12658.52685SRS1041036\_contig\_number\_contig-100\_7876.7877SRS1055034\_contig\_number\_contig-100\_17604.57776
25
186802
7

4
541000
family

4
1263
genus

41978
species

SRS014313\_contig\_number\_contig-100\_790.53371SRS058723\_contig\_number\_7573SRS077335\_contig\_number\_contig-100\_141.59846SRS1055049\_contig\_number\_contig-100\_145.97313
4

186803
family
14

1
species
39491

SRS142503\_contig\_number\_54115


SRS047014\_contig\_number\_38373
1898203
species
1

genus
841
12

12
2049040
species

SRS012969\_contig\_number\_37941SRS013951\_contig\_number\_41160SRS014412\_contig\_number\_contig-100\_831.124083SRS015431\_contig\_number\_82054SRS015579\_contig\_number\_16054SRS019068\_contig\_number\_85527SRS019161\_contig\_number\_27239SRS050941\_contig\_number\_contig-100\_507.138315SRS075878\_contig\_number\_contig-100\_192.154153SRS097889\_contig\_number\_39168SRS143780\_contig\_number\_32848SRS149784\_contig\_number\_27637
